# *Porphyromonas gingivalis*-OMVs promote the epithelial-mesenchymal transition of oral squamous cell carcinoma by inhibiting ferroptosis through the NF-κB pathway

**DOI:** 10.1080/20002297.2025.2482924

**Published:** 2025-04-03

**Authors:** Xinyue Liao, Hang Si, Yongxian Lai, Xiaoyan Zhang, Yun Feng, Tiejun Zhou, Yan Feng, Li Yu

**Affiliations:** a Department of Pediatric Dentistry, The Affiliated Stomatology Hospital of Southwest Medical University, Luzhou, China; b Oral & Maxillofacial Reconstruction and Regeneration of Luzhou Key Laboratory, Luzhou, China; c School of Stomatology, Southwest Medical University, Luzhou, China; d Department of Preventive Health Care, The Affiliated Stomatology Hospital of Southwest Medical University, Luzhou, China; e Department of Periodontics & Oral Mucosal Diseases, The Affiliated Stomatology Hospital of Southwest Medical University, Luzhou, China; f Department of Pathology, The Affiliated Hospital of Southwest Medical University, Luzhou, China; g NHC Key Laboratory of Nuclear Technology Medical Transformation (Mianyang Central Hospital), Mianyang, China

**Keywords:** *Porphyromonas gingivalis*, outer membrane vesicles, ferroptosis, epithelial-mesenchymal transition, oral squamous cell carcinoma

## Abstract

**Background:**

Recent studies reported the role of *Porphyromonas gingivalis* (*P. g*) in promoting oral squamous cell carcinoma (OSCC) progression. However, the molecular mechanism remains unclear.

**Materials and methods:**

*P. g*-OMVs were isolated using ultracentrifugation method and characterized by transmission electron microscopy (TEM) and nanoparticle tracking analysis (NTA). CCK-8, migration, invasion, Quantitative real-time Polymerase Chain Reaction (qRT-PCR) and immunocytochemistry assays were performed to evaluate the effect of *P. g*-OMVs on tumor cells’ proliferation, migration, invasion, epithelial-mesenchymal transition (EMT), and ferroptosis in vitro. Western blot was performed to study the phosphorylation of transcription factor nuclear factor kappa B (NF-κB). In vivo, the effect of *P. g*-OMVs on the growth of OSCC was evaluated using a xenograft tumor model, followed by hematoxylin and eosin and immunohistochemistry staining.

**Results:**

TEM and NTA demonstrated that *P. g*-OMVs have a vesicular structure with a particle size of around 118 nm. Compared to the control group, *P. g*-OMVs significantly enhance the proliferation, migration, and invasion of tumor cells. In addition, *P.* *g*-OMVs promote the EMT of OSCC cells, which can be attenuated by ferroptosis activator erastin. Moreover, *P. g*-OMVs inhibit feroptosis of OSCC by activating NF-κB signaling. In vivo, *P. g*-OMVs significantly enhance tumor growth of OSCC. Inhibition of NF-κB could significnatly reduce the growth of OSCC, which can be further rescued using ferroptosis inhibitor Ferrostain-1.

**Conclusions:**

*P. g*-OMVs promote OSCC progression by modulating the ferroptosis-related EMT through NF-κB signaling.

## Introduction

Oral squamous cell carcinoma (OSCC) is the most common malignant epithelial cancer in the maxillofacial region, with more than 370,000 new cases and 170,000 deaths detected in 2020 [1]. The 5-year overall survival of OSCC is under 50% due to its progressive invasion [2]. Therefore, it is urgent to identify the risk factors that facilitate the progression of OSCC.

The oral cavity is the second largest microbial habitat in the human body next to the gut [3]. Under normal circumstances, the oral microbiome maintains a dynamic equilibrium. However, dysbiosis of the oral microbiome leads to various infectious oral diseases, with periodontitis being the most common [4]. Several studies have pointed out that periodontitis is involved in the progression of a wide range of systemic diseases including cardiovascular diseases [5], diabetes [6], Alzheimer’s disease [7], and cancers [8]. Periodontitis is one of the risk factors for the progression of tumors such as head and neck cancer [9], lung cancer [10], breast cancer [11], and digestive tract cancer [12]. *Porphyromonas gingivalis* (*P. g*), the major pathogen of periodontitis, has been demonstrated to be detected in numerous tumor tissues [13,14]. In 2022, Dr. Hanahan updated the hallmarks of cancer, including one of the new hallmarks polymorphic microbiomes, which emphasized diversity and functional regulation of the microbiomes in the malignant progression of cancer [15]. *P. g* activates the host immune system and induces a sustained inflammatory response by releasing virulence factors such as lipopolysaccharides, gingipains, and other toxins to increase the proliferation and invasion of tumor cells [16]. *P. g* promotes the proliferation of OSCC by enhancing the cyclin D1 expression through the miR-21/programmed cell death 4/activator protein 1-negative feedback signaling pathway [17]. Moreover, continuous *P. g* infection increases the invasiveness of OSCC cells by markedly increasing the expression of interleukin (IL)-8 and matrix metalloproteinases (MMPs) [18]. In addition, Chunrong et al. [19] found that the amount of *P. g* was positively correlated with advanced clinical staging, poor differentiation, and lymph node metastasis in patients with OSCC. Furthermore, a high abundance of *P. g* in oral cancer tissues was reported to activate the expression of epithelial-mesenchymal transition (EMT)-related transcription factors such as snail and zinc-finger E-box binding protein 1, which, in turn, suppressed the expression of epithelial marker E-cadherin (E-cad) and upregulated the levels of mesenchymal markers N-cadherin (N-cad), MMP9, and Vimentin to promote the EMT of OSCC [20,21]. However, the specific communication mechanisms of *P. g* in promoting OSCC need to be investigated.

Outer membrane vesicles (OMVs) are 30–150 nm vesicles secreted by gram-negative microbiome with a lipid bilayer membrane structure [22]. OMVs transport a variety of biologically active molecules including protein, lipid, mRNA, miRNA, DNA, and long non-coding RNA into the receptor cells, to be involved in controlling the pathological processes of the receptor cells [23,24]. A single *P. g* secretes approximately 2,000 OMVs, suggesting that OMVs may promote the pathogenesis of diseases more competently compared with their parent microbiome [25]. Previous evidence has demonstrated that exosomes secreted by tumor cells orchestrate the cancer progression by modulating vascular leakage, angiogenesis, immune response, and metabolic reprogramming [26]. In recent years, the involvement of microbiome OMVs in intercellular communication has been suggested [27]. *Helicobacter pylori* (*H. p*)-OMVs promote the EMT phenotype of hepatic stellate cells by down-regulating E-cad and up-regulating Vimentin expression [28]. In addition, *H. p*-OMVs accelerate gastric cancer progression by stimulating the release of oncostatin M and activating its receptor expression in macrophages [29]. *Fusobacterium nucleatum* (*F. n*)-OMVs promote lung metastasis by activating autophagy in OSCC cells [30]. Importantly, functional diversity of *P. g*-OMVs in inducing systemic diseases including periodontitis [31], diabetes [32], cardiovascular diseases [33], and tumors [34] has been reported. Therefore, it is imperative to explore the mechanism of *P. g*-OMVs in facilitating the progression of OSCC. Ferroptosis is an iron-dependent cell death caused by iron overload, lipid peroxidation, and dysfunction of cellular antioxidant systems [35]. A recent study showed that *P. g* could provoke ferroptosis in hepatocytes in vitro and in the liver in vivo by inducing the Th17/Treg imbalance [36]. Numerous studies have demonstrated that ferroptosis mediates tumor progression by modulating EMT, angiogenesis, invasion, and metastasis [37]. EMT, a process in which tumor cells lose the tight junctions between epithelial cells and transform into a mesenchymal cell phenotype, is an integral hallmark of tumor metastasis. High Bach1 expression promotes EMT in glioma cells by mediating ferroptosis sensitivity in tumor cells, while ferroptosis activator efficiently transforms tumors from an invasive phenotype to an inhibitory phenotype [38]. *P. g* disrupts the oral epithelial barrier by inducing ferroptosis through inhibition of the solute carrier family 7 member 11 (SLC7A11)/glutathione/glutathione peroxidase 4 (GPX4) axis [39]. However, whether *P. g*-OMVs regulate the EMT of OSCC via ferroptosis requires further investigation.

In the current study, we examined the effect of *P. g*-OMVs on cellular mobility, EMT, and ferroptosis of tumor cells using 2D and 3D cell culture models in vitro. In addition, we determined the contribution of *P. g*-OMVs to tumor growth using in vivo xenograft tumor models.

## Materials and methods

### P. g culture and P. g-OMVs identification

*P. g* (American Type Culture Collection (ATCC), BAA-308) was purchased from the Beijing Microbiological Culture Collection Center. To exclude the effect of heme chloride and vitamin K1 on the isolation of *P. g*-OMVs, *P. g* was anaerobically cultured in brain–heart infusion (BHI) medium (HKM, Guangdong, China 028,360) without the supplement of heme chloride and vitamin K1 at 37°C under 80% N_2_, 10% H_2_, and 10% CO_2_ conditions. *P. g*-OMVs were extracted from freshly cultured *P. g* using the ultracentrifugation method at 4°C. Briefly, the culture supernatant of *P. g* was obtained by centrifugation at 500 g for 10 min, followed by centrifugation at 2,000 g for 10 min. Then, the supernatant was filtered through 0.22 μm Millipore filters to exclude contaminants and further centrifuged at 175,000 g for 1.5 h twice to pellet the *P. g*-OMVs. *P. g*-OMVs were suspended in the phosphate-buffered saline (PBS) and stored at −80°C for further in vitro and in vivo use. The concentration of *P. g*-OMVs was determined using the BCA protein assay kit (Solarbio, Beijing, China, PC0020). The characterization of *P. g*-OMVs was performed using transmission electron microscopy (TEM) and nanoparticle tracking analysis (NTA).

### Cell lines and culture conditions

CAL-27 and SCC-9 human OSCC cell lines were obtained from the ATCC (Manassas, USA). CAL-27 and SCC-9 cells were cultured in Dulbecco’s Modified Eagle Medium (DMEM, Procell, Wuhan, China, PM150210) and DMEM/F12 (Procell, Wuhan, China, PM150312) supplemented with 10% heat-inactivated fetal bovine serum (FBS) and 1% penicillin/streptomycin antibiotic (Beyotime, Sichuan, China, C0222) at 37°C under 5% CO_2_ condition, respectively.

### Cell proliferation assay

CAL-27 and SCC-9 cells were inoculated in 96-well plates (5 × 10^3^ cells per well) and cultured for overnight. *P. g*-OMVs at the concentrations of 3 μg/ml, 5 μg/ml, and 10 μg/ml were added in each group (PBS was used as control) and the cell proliferation was measured after 0 h, 24 h, and 48 h of *P. g*-OMVs treatment by adding 10 µl of Cell Counting Kit-8 solution (CCK-8, Oriscience, Chengdu, China, CB101) to each well and incubate for another 3 h. Then, the Tecan Spark Enzyme Labeler (Biotek, Beijing, China) was used to measure the optical density (OD) of each well at 450 nm.

### Tumor cell migration and invasion assays

For the transwell migration assay, CAL-27 and SCC-9 cells were placed at a density of 10^5^ cells per transwell insert and cultured in 5% FBS and antibiotic-free DMEM medium. Followed by adding 5 ug/ml *P. g*-OMVs to the upper compartment, while the culture medium supplemented with 10% FBS was added to the lower compartment. After 24 h incubation, non-migrated cells were wiped with a cotton swab, migrated cells were fixed with 4% paraformaldehyde (PFA) for 10 min and stained with 0.5% crystal violet solution. The number of migrated cells in each group was photographed. For the transwell invasion assay, the upper chamber membrane was pre-coated with Matrigel (YEASEN, 40183ES08) evenly, followed by the protocol identically as the transwell migration assay. Invaded cells were stained and counted after 48 h invasion.

For 3D cell migration and invasion assays, 3D cells were prepared. Briefly, CAL-27 and SCC-9 cells were seeded in a U-bottom non-adhesive 96-well plate and cultured for 24 h to obtain the 3D cell, the success of the 3D cell was confirmed by monitoring a single sphere in each well under the microscope. Then, for the 3D cell migration assay, one 3D cell was transferred into each well of a flat-bottom 96-well plate, *P. g*-OMVs (5 ug/ml) were added per well (PBS as a control), and the number of cells expanding from the cell 3D cell was dynamically monitored under a microscope and quantified. For the 3D cell invasion assay, the flat-bottom 96-well plate was coated using Matrigel at 37°C for 2 h, followed by transferring 3D cell into each well of the Matrigel-coated flat-bottom 96-well plate. The following protocol was identical to the 3D cell migration assay. Invaded cells were imaged and quantified using Image J.

### Hematoxylin and eosin (H&E) staining

For cell H&E staining, CAL-27 and SCC-9 cells seeded on the glass coverslips were treated with *P. g-*OMVs or the corresponding reagents for the indicated time and fixed with 4% PFA. For the tissue H&E staining, 4-μm-thick paraffin-embedded mouse tumor tissues were stained using H&E staining to evaluate their pathological characteristics. Images were captured with a BX-43 upright optical microscope.

### Immunocytochemistry (ICC)

Glass coverslip cultured CAL-27 and SCC-9 cells were stimulated with *P. g-*OMVs or the corresponding reagents for the indicated time followed by fixation with 4% PFA. Then, the cells were stained with the primary anti-Ki67 (Bioss, Beijing, China), E-cad (Bioss, Beijing, China), Vimentin (Abmart, Shanghai, China), and GPX4 antibodies (Abmart, Shanghai, China), followed by incubation with goat anti-rabbit IgG (H+L) HRP secondary antibody (Affinity, Jiangsu, China) and counterstained with DAB (3, 3’−diaminobenzidine), or incubation with goat anti-rabbit IgG (H+L) Alexa Fluor 594 secondary antibody (Oriscience, Sichuan, China) or goat anti-rabbit IgG (H+L) Alexa Fluor 488 secondary antibody (Oriscience, Sichuan, China), and counterstained with DAPI. The DAB staining images were photographed under the BX-43 upright optical microscope, and immunofluorescence images were captured under the BX-53 orthogonal fluorescence microscope. The positive areas were quantified using ImageJ software, 5 images per group.

### Immunohistochemistry (IHC)

Formalin-fixed, paraffin-embedded mouse tumor tissues were deparaffinized, rehydrated, followed by antigen retrieval. Then, tissues were blocked with 5% goat serum, and incubated with an anti-Ki67 antibody overnight. Goat anti-rabbit IgG (H+L) HRP secondary antibody was used the next day and counterstained by hematoxylin. The images were photographed by the BX-43 upright optical microscope and the integrated optical density (IOD) was analysed using ImageJ software.

### Quantitative real-time polymerase chain reaction (qRT-PCR)

The total RNA from cells was extracted by SteadyPure Quick RNA Extraction Kit (Accurate biology, Hunan, China, AG21023) and synthesized into cDNA using Evo M-MLV RT Kit (Accurate biology, Hunan, China, AG11707). The cDNA was utilized for the qRT-PCR using SYBR Green Premix Pro Taq HS qPCR Kit (Accurate biology, Hunan, China, AG11701) in accordance with the instructions. The relative mRNA expression levels were calculated using the 2^−∆∆Ct^ method with GAPDH used as an internal control. The sequences of the primers are shown in the supplementary Table S1.

### Western blot analysis

CAL-27 and SCC-9 cells treated with or without *P. g*-OMVs were lysed, and the total protein was examined using the BCA protein assay kit. Western blot analysis was conducted using primary anti-Phospho-NF-κB p65 (Ser536) (93H1) (Cell Signaling, Massachusetts, USA, 3033), NF-κB p65 (D14E12) (Cell Signaling, Massachusetts, USA, 8241) and β-actin (Bioss, Beijing, China) along with the corresponding goat anti-rabbit IgG (H+L) HRP secondary antibody. Protein bands were visualized using the ECL kit (Solarbio, Beijing, China, PE0010) and their intensity was measured using ImageJ software.

### Ferroptosis activator treatment

In vitro, CAL-27 and SCC-9 cells were resuspended in serum-free and antibiotic-free medium and placed in 6-well plates. Then, three groups (1) PBS group, (2) *P. g*-OMVs group, and (3) *P. g*-OMVs + erastin group were divided, followed by the corresponding treatment. Briefly, cells were treated with *P. g*-OMVs (5 ug/ml) for 3 h, followed by 10 μM ferroptosis activator erastin (Mackin, Shanghai, China, E872563) treatment for another 48 h. Further, RNA was isolated, and the mRNA expressions of E-cad, N-cad, and Vimentin were evaluated using qRT-PCR. Cells were fixed and ICC staining was performed to evaluate the protein expressions of E-cad and Vimentin.

### Transcription factor nuclear factor kappa B (NF-κB) inhibition

2 × 10^5^ cells/well of CAL-27 and SCC-9 cells were placed in 6-well plates. Then, three groups (1) PBS group, (2) *P. g*-OMVs group, and (3) *P. g*-OMVs + NF-κB inhibitor BAY 11–7082 (MCE, New Jersey, USA 19,542-67-7) group were divided, followed by the corresponding treatment. The cells were pretreated with 10 μM BAY 11–7082 for 1 h, then stimulated with *P. g*-OMVs for 48 h (PBS was used as a control). Then, RNA was extracted and qRT-PCR was used to assess the mRNA expressions of the GPX4, solute carrier family 7 member 11 (SLC7A11), prostaglandin-endoperoxide synthase 2 (PTSG2), and transferrin receptor (TFR).

### In vivo mouse model and treatment

5 × 10^6^ SCC-9 cells were subcutaneously injected into the right flank of BALB/c nude mice (female, 18–20 g, 6–7 weeks old) to establish a tumor xenograft model. Tumor volume was measured on a 4-day basis according to the following formula: V (mm^3^) = 0.5 × length × width^2^. The mice were randomly divided into four groups, including (1) control group, (2) *P. g*-OMVs group, (3) *P. g*-OMVs + BAY 11–7082 group, and (4) *P. g*-OMVs + BAY 11–7082 + Ferrostain-1 (Fer-1) group, when the tumors reached about 100 mm^3^ in volume. Then, the treatment of *P. g*-OMVs, BAY 11–7082, and ferroptosis inhibitor Fer-1 was started. Briefly, *P. g*-OMVs (1 mg/kg) were injected intratumorally into the tumor-bearing mice once a week (PBS was injected as a control), BAY 11–7082 (2.5 mg/kg/twice a week for 3 weeks, intraperitoneally) was injected (DMSO was injected as a control), Fer-1 (0.655 mg/kg/three times a week for 3 weeks, intraperitoneally, MCE, New Jersey, USA, HY-100579) was administered (DMSO was injected as a control). Mice were sacrificed 31 days after the tumor cell implantation, and tumors were dissected and weighed. Then, the tumor tissues were embedded in paraffin or optimal cutting temperature (OCT) compound, followed by H&E staining and IHC. All mouse experiments were performed with approval and under the approval and supervision of the Experimental Animal Ethics Committee of Southwest Medical University (NO: SWMU20240089).

### Statistical analysis

All data were presented as mean ± standard deviation (SD) of three independent experiments. GraphPad Prism 9.0 software was used for statistical analysis, Student t-test was used for significance comparison between two groups, and one-way or two-way analysis of variance (ANOVA) was used for significance identification between multiple groups. Differences were considered statistically significant at *P*< 0.05 and indicated with one asterisk.

## Results

### P. g-OMVs promote the proliferation of OSCC

**Figure 1.**
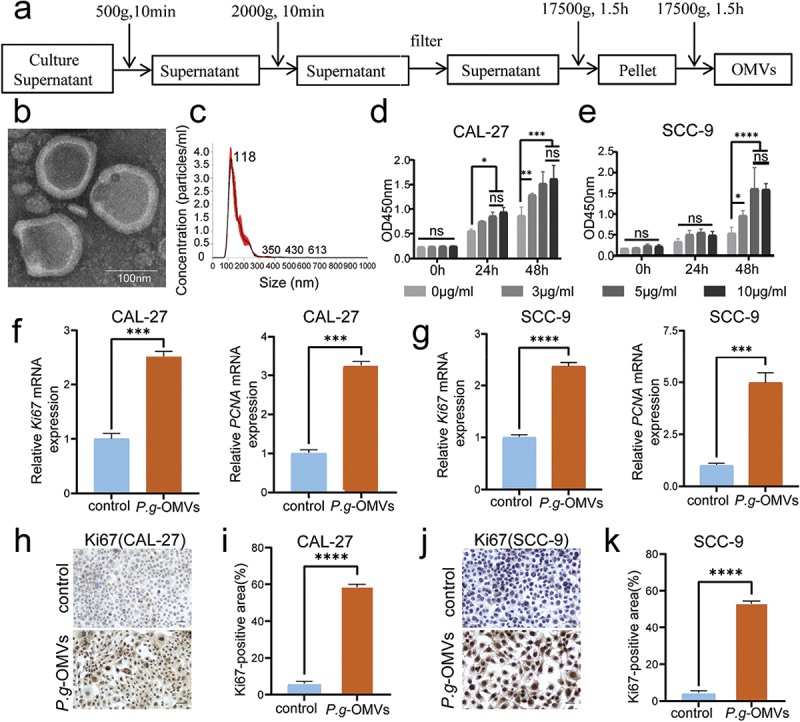
*P. g*-OMVs promote the proliferation of OSCC. (a–c) Extraction and identification of *P. g*-OMVs; (a) The schematic showed the steps of *P. g*-OMVs extraction; (b) Morphology of *P. g*-OMVs observed by TEM (scale bars: 100nm); (c) The particle size of *P. g*-OMVs was distributed by NTA. (d, e) OD value of CAL-27 and SCC-9 cells after stimulated with *P. g*-OMVs at different concentrations and different stimulation times, *n*=6. (f, g) The expression levels of Ki67 and PCNA mRNA in CAL-27 (f) and SCC-9 (g) cells were evaluated using qRT-PCR after stimulation with *P. g*-OMVs, *n*=3 real-time PCR replicates. (h-k) The ICC staining of Ki67; representative images of Ki67 in CAL-27 (h) and SCC-9 (j) cells with *P. g*-OMVs treatment (scale bars: 100μm); the Ki67-positive areas of CAL-27 (I) and SCC-9 (k) cells were quantified respectively, *n*=5 fields. Data represent the mean±SD; ns, not significant; *p < 0.05, **p < 0.01, ***p < 0.001, ****p < 0.0001; Two-way ANOVA (d, e) and student’s t-test (f, g, i, k) were used.

The *P. g*-OMVs were isolated from the culture supernatant of *P. g* using ultracentrifugation method (Figure 1a). The TEM demonstrated the round or elliptical vesicle-like bilayer structures with a uniform diameter of approximately 100 nm of the *P. g*-OMVs (Figure 1b). NTA demonstrated that the particle size of *P. g*-OMVs was predominantly distributed at 118 nm (Figure 1c). The results of TEM and NTA suggested the successful extraction of *P. g*-OMVs. To investigate the effect of *P. g*-OMVs on the proliferation of OSCC cells, we selected poor-differentiated, aggressive and highly malignant SCC-9 cells and well-differentiated, low malignant CAL-27 cells to verify the generalizability of the findings. We conducted a cell proliferation assay and measured the elevated proliferation of CAL-27 (Figure 1d) and SCC-9 (Figure 1e) cells after co-cultured with *P. g*-OMVs in both time-dependent and concentration-dependent manners, indicating that *P. g*-OMVs greatly promote the proliferation of OSCC cells. However, no proliferation difference was detected between 5 μg/ml and 10 μg/ml *P. g*-OMVs at different time points (Figure 1d,e), consequently, 5 μg/ml of *P. g*-OMVs was selected as the experimental concentration for subsequent cell biology assays. Next, qRT-PCR detected the markedly increased expression of the proliferative nuclear proteins Ki67 and PCNA in CAL-27 (Figure 1f) and SCC-9 (Figure 1g) cells with *P. g*-OMVs stimulation. Additionally, ICC measured the elevated expression of Ki67 protein level in CAL-27 (Figure 1h,i) and SCC-9 (Figure 1j,k) cells after treated with *P. g*-OMVs. These findings indicated that *P. g*-OMVs effectively promote the proliferation of OSCC cells.

### P. g-OMVs enhance the migration and invasion abilities of OSCC cells

**Figure 2.**
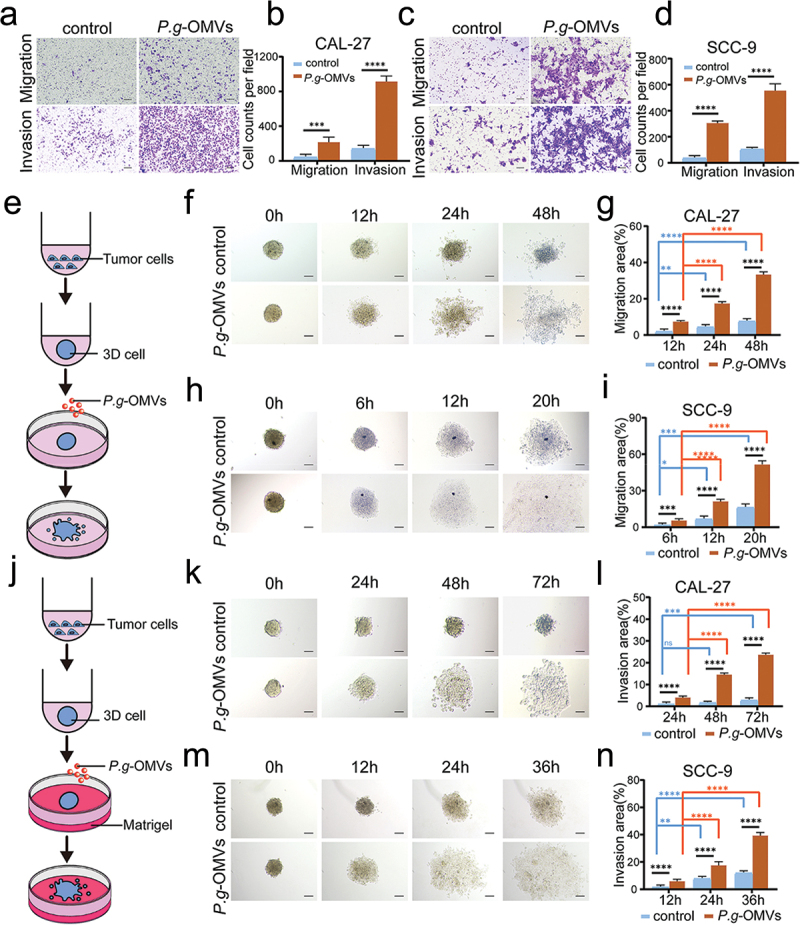
*P. g*-OMVs enhance the migration and invasion abilities of OSCC cells. (a–d) The effect of *P. g*-OMVs on the migration and invasion abilities of CAL-27 and SCC-9 cells were tested using transwell assays without or with Matrigel coating, respectively. The CAL-27 (a) and SCC-9 (c) cells migrating (a, c upper) and invading (a, c lower) through the membrane were photographed (scale bars: 100μm) and counted (b, d), *n*=5 fields. (e, j) Schematic diagram of 3D cell migration and invasion. (f–i) The 3D cell migration assay was performed to study the migration of OSCC cells; The representative images of CAL-27 (f) and SCC-9 (h) cells migrating out of the 3D cell were photographed (scale bars: 100μm); the migrated cells were counted (g, i), *n*=5 fields. (k–n) 3D cell invasion assay was utilized to evaluate the invasion of OSCC cells; The CAL-27 (k) and SCC-9 (m) cells invaded out of the 3D cell were photographed (scale bars: 100μm) and counted (l, n), *n*=5 fields. Data represent the mean±SD; ns, not significant; *p < 0.05, **p < 0.01, ***p < 0.001, ****p < 0.0001; Student’s t-test (b, d) and two-way ANOVA (g, i, l, n) were used.

To study the contribution of *P. g*-OMVs to cell mobilities, transwell migration and invasion assays were performed. The results indicated that the migration (24 h) and invasion (48 h) of CAL-27 (Figure 2a,b) and SCC-9 (Figure 2c,d) cells were enhanced after stimulation with *P. g*-OMVs. To reflect a more accurately in vivo environment, we employed 3D cell culture models to dynamically observe the tumor cells’ movement by investigating the number of cells climbing out of the 3D cell (Figure 2e,j). With the prolonged time, the number of CAL-27 cells that migrated out of the 3D cell was significantly enhanced in the *P. g*-OMVs group as compared to the control group (Figure 2f,g). A similar result was achieved in SCC-9 cells except the micrographs collection ends at 20 h (Figure 2h,i). Additionally, the invasion of CAL-27 cells stimulated by *P. g*-OMVs was statistically enhanced as compared to the control group, with the images photographed at 0 h, 24 h, 48 h, and 72 h (Figure 2k,l). The significant invasion of SCC-9 cells was evaluated with image photography ends at 36 h (Figure 2m,n). These findings suggested that *P. g*-OMVs promote the migration and invasion of OSCC cells in vitro, and SCC-9 cells demonstrated superior migration and invasion capabilities than CAL-27 cells.

### P. g-OMVs promote the EMT progression in OSCC cells

**Figure 3.**
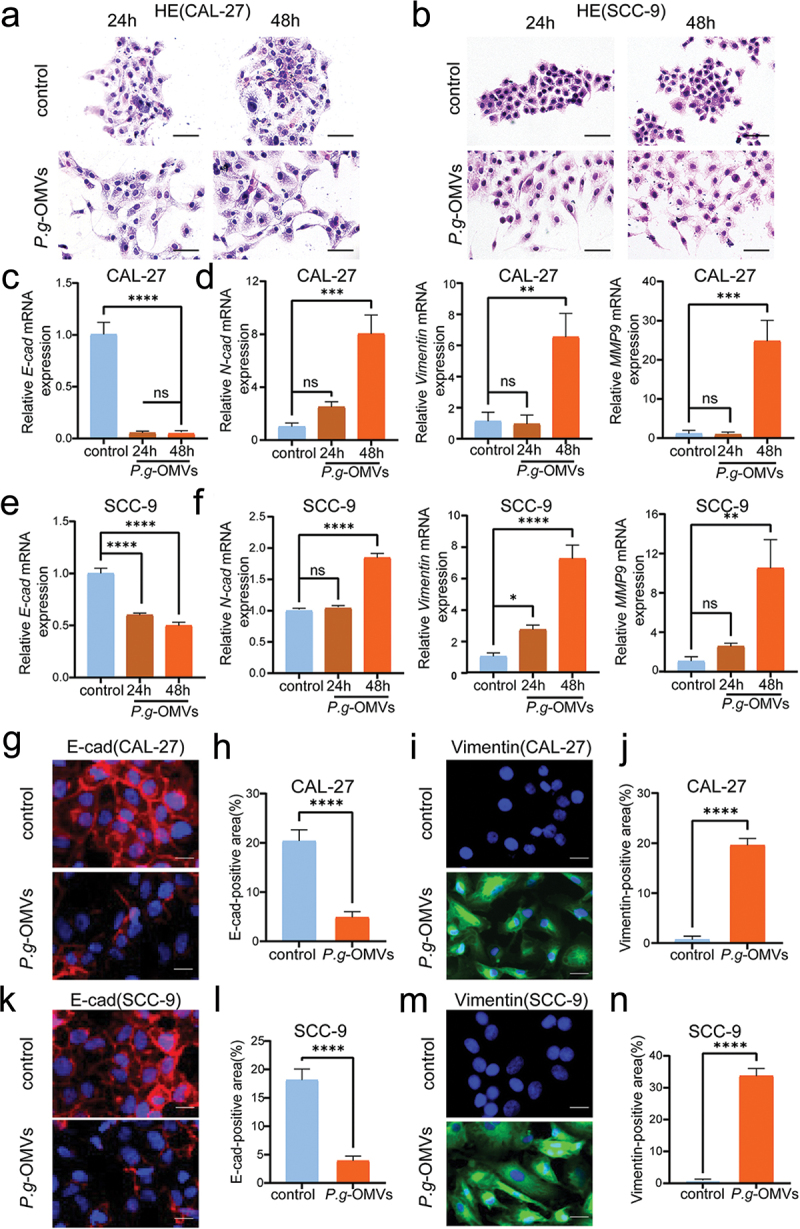
*P. g*-OMVs promote the EMT progression in OSCC cells. (a, b) Typical images of CAL-27 (a) and SCC-9 (b) cells stained with H&E (scale bars: 50μm), *n* = 5 fields. (c–f) E-cad, N-cad, Vimentin, and MMP9 mRNA expressions in CAL-27 (c, d) and SCC-9 (e, f) cells were detected using qRT-PCR after treated with *P. g*-OMVs, *n* = 3 real-time PCR replicates. (g–m) The OSCC cells were stained with E-cad and Vimentin using immunofluorescence staining; typical images of CAL-27 stained with E-cad (g) and Vimentin (i) were photographed (scale bars: 100μm); and the positive areas of E-cad (h) and Vimentin (j) were quantified, representative images of SCC-9 stained with E-cad (k) and Vimentin (m) were photographed (scale bars: 100μm); and the positive areas of E-cad (l) and Vimentin (n) were quantified, *n* = 5 fields. Data represent the mean±SD; ns, not significant; *p < 0.05, **p < 0.01, ***p < 0.001, ****p < 0.0001; One-way ANOVA (c-f) and student’s t-test (h, j, l, n) were used.

EMT is the initial step when cancer cells acquiring metastasis ability. Since *P. g*-OMVs promote the migration and invasion of OSCC cells, we wonder whether *P. g*-OMVs treatment influences the morphology changes of OSCC cells. Therefore, we performed H&E staining and evaluated the morphological changes of OSCC cells. CAL-27 (Figure 3a) and SCC-9 (Figure 3b) cells changed from regular polygonal shape to spindle shape, similar to the morphological characteristics of mesenchymal cells after *P. g*-OMVs treatment. Therefore, we hypothesize that *P. g*-OMVs may be involved in generating the EMT of OSCC cells. qRT-PCR measured the expressions of the EMT markers E-cad, N-cad, Vimentin, and MMP9. The results showed that the expression of epithelial marker E-cad (Figure 3c,e) was decreased and the expressions of mesenchymal markers N-cad, Vimentin, and MMP9 (Figure 3d,f) were increased in CAL-27 (Figure 3c,d) and SCC-9 (Figure 3e,f) following stimulation with *P. g*-OMVs. Consistently, the protein level of E-cad (Figure 3g,h,k,l) was decreased, and the expression of Vimentin (Figure 3i,j,m,n) was increased in CAL-27 (Figure 3g–j) and SCC-9 (Figure 3k–n) cells with *P. g*-OMVs stimulation. These results indicated that *P. g*-OMVs promoted the EMT of OSCC cells.

### P. g-OMVs inhibit the ferroptosis sensitivity of OSCC cells through the NF-κB pathway

**Figure 4.**
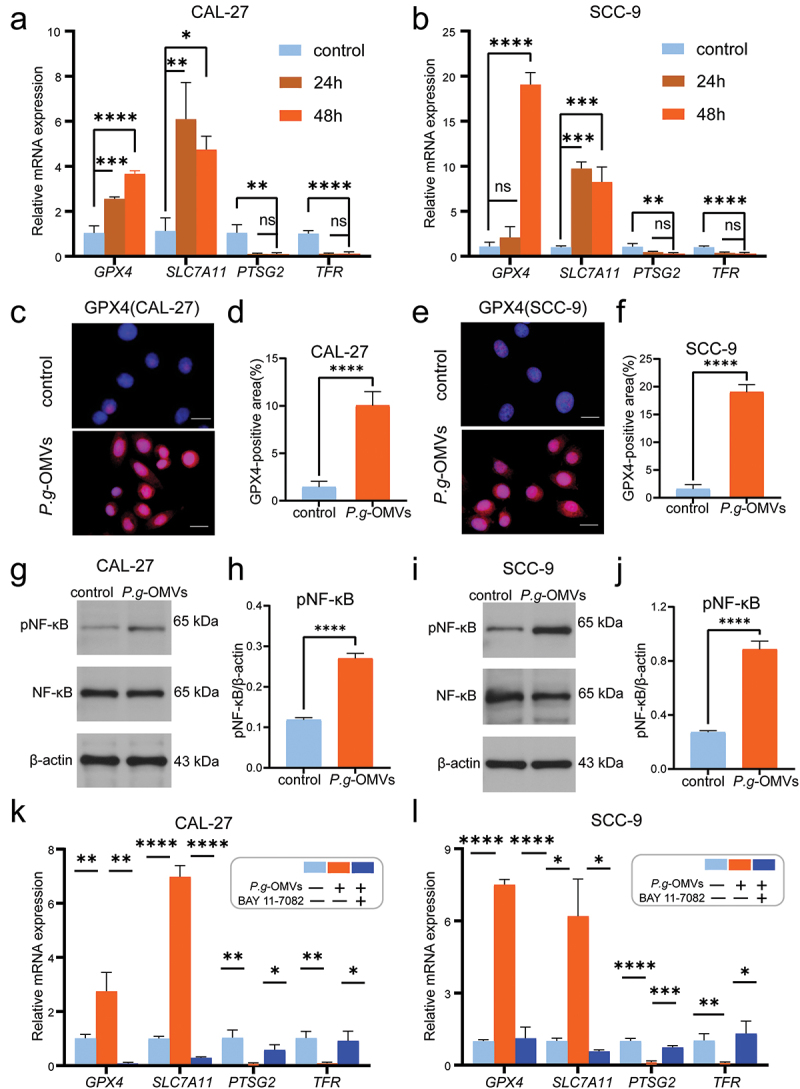
*P. g*-OMVs inhibit the ferroptosis sensitivity of OSCC cells through the NF-κB pathway. (a, b) GPX4, SLC7A11, PTSG2, and TFR mRNA expressions in CAL-27 (a) and SCC-9 (b) cells were detected using qRT-PCR after treatment with *P. g*-OMVs, *n* = 3 real-time PCR replicates. (c–f) The OSCC cells were stained with GPX4 using immunofluorescence staining; typical images of CAL-27 (c) and SCC-9 (e) cells were photographed (scale bars: 100μm); and the positive area of GPX4 in CAL-27 (d) and SCC-9 (f) cells was quantified, *n* = 5 fields. (g–j) The expressions of phospho-NF-κB and NF-κB protein in CAL-27 (g) and SCC-9 (I) cells were determined by western blot after treatment with *P. g*-OMVs; and the densities in CAL-27 (h) and SCC-9 (j) cells were quantified. (k, l) Cells were stimulated with BAY 11–7082 for 1h, followed by *P. g*-OMVs stimulation; GPX4, SLC7A11, PTSG2, and TFR mRNA expression levels in CAL-27 (k) and SCC-9 (l) cells were assessed by qRT-PCR, *n* = 3 real-time PCR replicates. Data represent the mean±SD; ns, not significant; *p < 0.05, **p < 0.01, ***p < 0.001, ****p < 0.0001; One-way ANOVA (a, b, k, l) and student’s t-test (d, f, h, j) were used.

The development and treatment of OSCC are closely associated with the process of ferroptosis. It has been demonstrated that the migration and invasion ability of OSCC cells could be promoted by suppression of miR-615-5p through regulating SLC7A11-mediated ferroptosis [40]. In this study, the qRT-PCR results confirmed that the mRNA expression levels of GPX4 and SLC7A11 were upregulated and the mRNA levels of PTSG2 and TFR were downregulated in CAL-27 (Figure 4a) and SCC-9 (Figure 4b) cells with *P. g*-OMVs stimulation. Collectively, the protein level of GPX4 was increased after *P. g*-OMVs stimulation in CAL-27 (Figure 4c,d) and SCC-9 (Figure 4e,f) cells, suggesting that *P. g-*OMVs may be involved in inhibiting ferroptosis sensitivity of OSCC cells. The NF-κB pathway is critical for regulating the inflammatory response and serves as a hallmark and therapeutic target for cancer progression [41]. A study found that SIRT6 may promote ferroptosis and inhibit glycolysis by suppressing the NF-κB pathway in pancreatic cancer [42]. Western blot analysis indicated the activation of NF-κB in *P. g*-OMVs group as compared to the control group, suggesting that *P. g*-OMVs could effectively stimulate phosphorylation of NF-κB in CAL-27 (Figure 4g,h) and SCC-9 (Figure 4i,j) cells. To explore whether the *P. g-*OMVs regulate the ferroptosis of OSCC cells through the NF-κB pathway, we pre-treated CAL-27 and SCC-9 cells with NF-κB inhibitor BAY 11–7082, followed by *P. g*-OMVs stimulation. qRT-PCR data showed that BAY 11–7082 attenuated the expression of GPX4 and SLC7A11, and increased the expressions of PTSG2 and TFR in CAL-27 (Figure 4k) and SCC-9 (Figure 4l) cells, which were differentially regulated following *P. g*-OMVs treatment. Based on the above data, we proposed that *P. g*-OMVs may inhibit ferroptosis of OSCC cells via the NF-κB pathway.

### P. g-OMVs induce EMT phenotype of OSCC cells by inhibiting the ferroptosis

**Figure 5.**
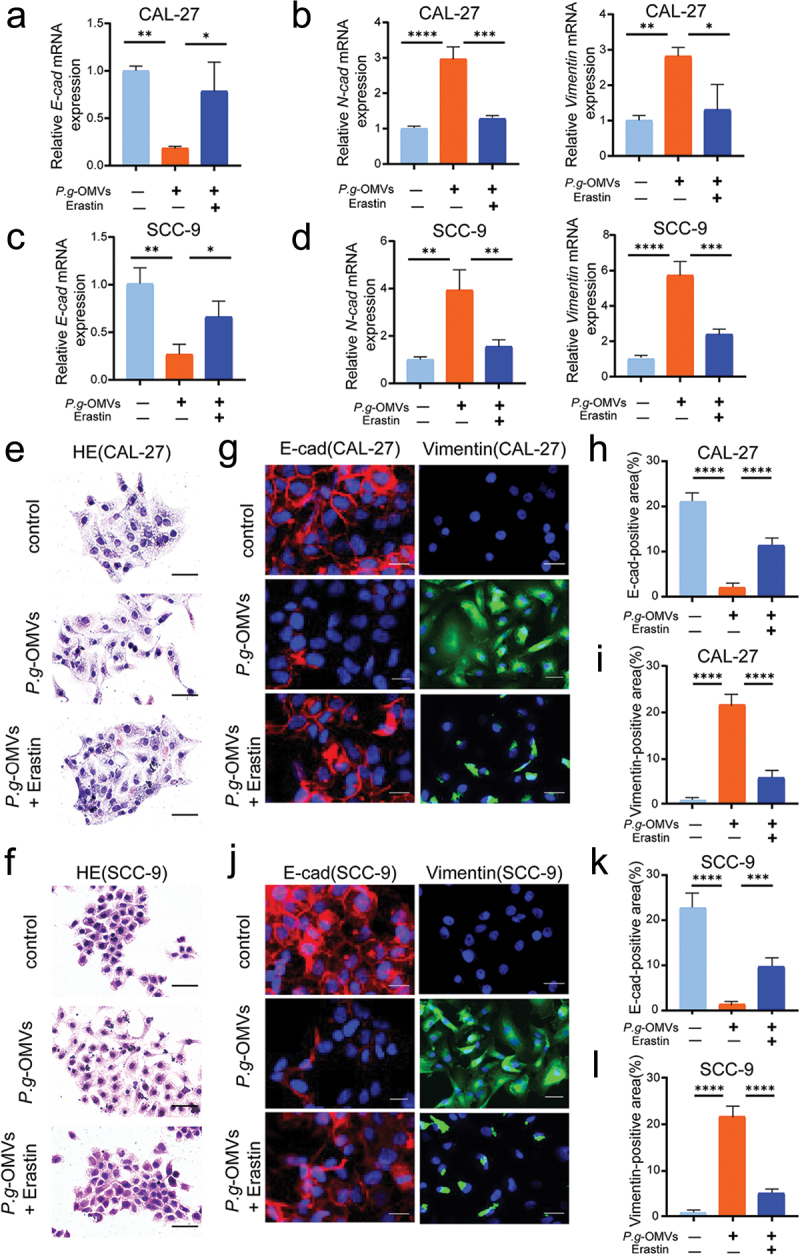
*P. g*-OMVs induce EMT phenotype of OSCC cells by inhibiting the ferroptosis. (a–d) Cells were stimulated with *P. g*-OMVs for 3h, followed by erastin treatment for 48h; and E-cad, N-cad, and Vimentin mRNA expressions in CAL-27 (a, b) and SCC-9 (c, d) cells were assessed by qRT-PCR, n = 3 real-time PCR replicates. (e, f) Typical images of CAL-27 (e) and SCC-9 (f) cells stained with H&E (scale bars: 50μm), n = 5 fields. (g–l) The OSCC cells were stained with E-cad and Vimentin; typical images of CAL-27 (g) and SCC-9 (J) cells were photographed (scale bars: 100μm); and the positive areas of E-cad (h, k) and Vimentin (i, l) of CAL-27 (h, i) and SCC-9 (k, l) cells were quantified, n = 5 fields. Data represent the mean±SD; *p < 0.05, **p < 0.01, ***p < 0.001, ****p < 0.0001; One-way ANOVA (a-d, h, i, k, l) was used.

To explore whether *P. g*-OMVs-induced EMT in OSCC cells is mediated by ferroptosis, the ferroptosis activator erastin was utilized. qRT-PCR showed that erastin reversed the expression of E-cad that was decreased by *P. g*-OMVs stimulation in CAL-27 (Figure 5a) and SCC-9 (Figure 5c); In addition, erastin reversed the expressions of N-cad, and Vimentin that were increased by *P. g*-OMVs stimulation in CAL-27 (Figure 5b) and SCC-9 (Figure 5d) cells. The morphological alterations in OSCC cells were examined using H&E staining. After treatment with *P. g*-OMVs and erastin, CAL-27 (Figure 5e) and SCC-9 (Figure 5f) cells exhibited an increase in intercellular adhesion, a transformation in cell morphology from spindle shape to regular polygonal shape was observed. At the protein level, erastin reversed the *P. g*-OMVs-mediated down-regulation of E-cad and up-regulation of Vimentin in CAL-27 (Figure 5g–i) and SCC-9 (Figure 5j–l) cells. As expected, these findings demonstrated that erastin inhibits *P. g*-OMVs-induced EMT of OSCC cells. In summary, our study provided convincing proof that *P. g*-OMVs induce EMT phenotype through ferroptosis thereby promoting the migration and metastasis of OSCC.

### P. g-OMVs promote the progression of OSCC through NF-κB regulated ferroptosis

**Figure 6.**
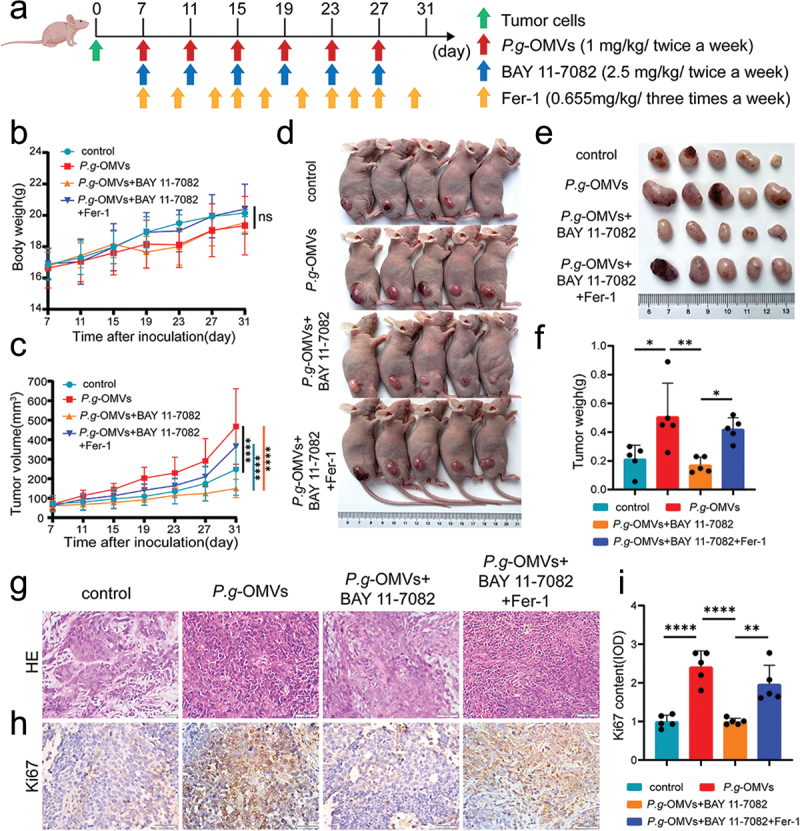
*P. g*-OMVs promote the progression of OSCC through NF-κB regulated ferroptosis. (a) Schematic diagram of the in vivo experiment. (b) The body weight index of nude mice. (c) The tumor volume index of nude mice. (d) Images of the tumor-bearing mice. (e) Photographs of tumors. (f) Weight of tumor samples. (g) Typical images of mouse tumor tissues stained with H&E (scale bars: 100μm). (h, i) The mouse tumor tissues were stained with Ki67, typical images were photographed (scale bars: 100μm) (h); and the positive areas of Ki67 were quantified (i), *n* = 5 fields. Data represent the mean±SD; = *p < 0.05, **p < 0.01, ****p < 0.0001; Two-way ANOVA (b, c) and one-way ANOVA (f, i) were used, five mice per group.

In order to evaluate whether the promotion of tumor growth by *P. g*-OMVs in vivo is regulated by ferroptosis via the NF-κB pathway, we subcutaneously injected SCC-9 cells into the right back of BALB/c nude mice. After the tumor volume reached approximately 100 mm^3^, *P. g*-OMVs (1 mg/kg body weight) were injected into the tumor tissue, Fer-1 (0.655 mg/kg body weight) and BAY 11–7082 (2.5 mg/kg body weight) were injected intraperitoneally in the following 24 days (Figure 6a). The body weight and tumor volume were measured every 4 days. Mice were sacrificed after 31 consecutive days of tumor injection. No significant difference in body weight was evaluated (Figure 6b). The tumor volume in the *P. g*-OMVs-treated group was significantly larger than that of the control group. However, the BAY 11–7082 treatment significantly inhibited the tumor growth, which can be reversed when co-implanted with BAY 11–7082 and Fer-1 (Figure 6c–f). Mouse tumor tissues were stained with H&E, and we found that the cells in the control group and the *P. g*-OMVs + BAY 11–7082 group were less dense, with cell breakage, abundant vacuolar structures, and cytoplasmic leakage, signs of cell necrosis such as nuclear fragmentation, cohesion, and lysis were much more prominent. In the *P. g*-OMVs group and the *P. g*-OMVs+ BAY 11–7082 + erastin group, the tumor cells were tightly-packed and well-defined with exuberant proliferation and deeply stained nuclei (Figure 6g). The expression of Ki67 in mouse tumor tissues was detected by IHC, and the results indicated that the expression level of Ki67 was upregulated in the *P. g*-OMVs group compared to the control group. After intervention with BAY 11–7082, the protein level of Ki67 was significantly reduced, suggesting that *P. g*-OMVs promote the OSCC growth through the NF-κB pathway. Consistent with in vitro experiments, we found that the Ki67 protein level in Fer-1 and BAY 11–7082 combination treatment group was increased compared to BAY 11–7082 group, which suggestted that *P. g*-OMVs may mediate ferroptosis through NF-κB pathway (Figure 6h,i). Together, we propose that *P. g*-OMVs inhibit ferroptosis through the NF-κB pathway to promote OSCC progression.

## Discussion and conclusion

In this study, we indicated that *P. g* promotes ferroptosis-related proliferation, migration, invasion, and EMT by modulating interactions with OSCC cells through the secretion of OMVs, thereby resulting in the malignant progression of OSCC. Our findings emphasize the clinical importance of oral hygiene maintenance in preventing the development of OSCC and other malignancies.

According to epidemiological studies, smoking and alcohol consumption are reported to be the main causative factors for OSCC. However, the incidence of OSCC in non-smokers and non-drinkers has increased significantly in recent years, suggesting that there are other promoting factors of OSCC [43]. In recent times, the homeostatic balance of the microbiome has become increasingly highlighted for its role in the etiology of various diseases, particularly in the progression of cancer [44]. Several reports have indicated that microbial dysbiosis contributes to the development of tumors such as pancreatic cancer [45], lung cancer [46] and colorectal cancer [47]. Therefore, identifying and controlling carcinogenic microbiomes could reduce the prevalence of tumors. Periodontitis is a chronic inflammatory disease caused by bacterial infection [48]. Periodontal pathogens include *P. g*, *Aggregatibacter actinomycetemcomitans* (*A. a*), *F. n*, and *Treponema denticola* [49]. A growing number of studies have shown that periodontal pathogens were associated with the malignant progression of various tumors [50]. A prospective cohort study found that high levels of *P. g* and *A. a* in tumor tissues were positively attributed to pancreatic cancer prevalence [51]. Higher levels of *F. n* were detected in esophageal cancer tissues as compared to normal tissues, implying that *F. n* may be one of the risk factors for the development of esophageal cancer [52]. Zhang et al. performed 16S rDNA microbiome sequencing of 50 OSCC patients and observed that the amount of oral microbiome such as *F. n, P. g*, and *A. a* in the tumor tissues was significantly higher than that in normal tissues, suggesting that periodontal pathogens play a key role in promoting OSCC progression [53]. Conventional therapy for OSCC includes tumor resection, radiotherapy, and chemotherapy. Radiotherapy and chemotherapy induce dysbiosis of the oral microbiome by forming an inflammatory microenvironment and damaging epithelial cells to suppress or damage the systemic immune system, resulting in an increase in the quantity and species of pathogens in patients with malignant tumors. Moreover, the accumulation of the pathogens could exacerbate pre-existing periodontal lesions and ultimately trigger osteonecrosis of the jaw; Whereas periodontal inflammation is the contributing factor for systemic infections [54,55]. There is a favorable correlation between the severity of periodontal disease and the prevalence of side symptoms such as mucositis, xerostomia, and dysgeusia in patients undergoing radiotherapy and chemotherapy [56]. A well-established basic periodontal therapy and plaque control prior to chemotherapy could significantly reduce the risk for febrile neutropenia [57]. Sato et al. demonstrated that oral hygiene care was beneficial in preventing post-operative infections in OSCC patients with surgical tumor resection [58]. Therefore, to reduce the risk of post-operative infection and tumor metastasis, therapy for microbiota infection diseases including dental caries and periodontitis prior to radiotherapy and chemotherapy is essential in reducing the incidence of microbiome infectious pneumonitis and other microbiota infections by reducing the accumulation of oral microbiome [59]. It was reported that *P. g*-OMVs were more pathogenic for periodontitis-associated systemic diseases than *P. g* [60]. Although the link between *P. g* and OSCC progression has been documented, whether *P. g* regulate the communication with OSCC cells by secreting OMVs remains elusive.

The effect of OMVs on tumor growth has attracted widespread attention. Early data demonstrated that *P. g*-OMVs significantly inhibit the proliferation of human gingival fibroblast in a dose-dependent manner, thereby generating chronic periodontitis [61]. Li et al. showed that *F. n*-OMVs stimulate the proliferation of breast cancer cells through Toll-like receptor 4 signaling [62]. Consistent with the previous observations, our results revealed that *P. g*-OMVs promote the proliferative capacity of OSCC cells (Figure 1d–k), and the same results were verified in the in vivo animal model (Figure 6). In addition, various reports have indicated the regulation role of OMVs in tumor migration and invasion. *F. n*-OMVs activated autophagy in tumor cells to enhance OSCC metastasis [30]. sRNA23392 packaged by *P. g*-OMVs promoted OSCC migration and invasion through desmocollin-2 signaling [63]. Consistently, our results demonstrated that *P. g*-OMVs promote the migration and invasion of OSCC cells by regulating EMT (Figure 2). Additionally, we observed that SCC-9 cells exhibit greater migration and invasion abilities than CAL-27 cells in both 2D and 3D cell models, as evidenced by the results that SCC-9 cells migrate and invade more when exposed to the same concentration of *P. g*-OMVs simultaneously. SCC-9 and CAL-27 cells differ in their degree of differentiation and malignancy. In line with earlier studies, the aforementioned data demonstrated that poor-differentiated SCC-9 cells possess stronger migration and invasion capacity than well-differentiated CAL-27 cells [64]. Ferroptosis, a type of atypical cell death differing from apoptosis and autophagy, is characterized by glutathione depletion and lipids peroxidation [65]. Dysregulation of ferroptosis results in various diseases including diabetic complications [66], neurodegenerative diseases [67], ischemia-reperfusion injury [68], and cancers [69]. A recent finding has revealed that IL-6 activate cystine glutamate reverses transporter(xCT) through the janus kinase 2/transcription 3 pathway to inhibit the ferroptosis of head and neck squamous cell carcinoma (HNSCC) thereby promoting tumor progression [70]. Tribbles homolog 3 enhances the malignancy of HNSCC by inhibiting ferroptosis through the attenuation of Fe^2+^ and cellular lipid peroxidation [70]. Therefore, inducing ferroptosis in tumor cells is a major clinical concern for clinicians. NF-κB pathway plays a crucial role in the regulation of inflammation, immune response, cell survival and apoptosis [41]. In addition, NF-κB activation could promote tumor progression by stimulating angiogenesis, promoting tumor cells proliferation, inducing EMT and facilitating tumor immune evasion [71]. NF-κB pathway has been shown to modulate oxidative stress and ferroptosis by regulating the transcription of antioxidant molecules such as heme oxygenase 1 and GPX4 [72]. It has also been elucidated that RAS-selective lethal enzyme 3 could regulate glioblastoma cell ferroptosis through the NF-κB pathway [73]. Alawylenolide II induces the ferroptosis of hepatocellular carcinoma cells by blocking the NF-κB pathway to inhibit tumor growth [74]. Previous studies demonstrated that *P. g* could activate NF-κB pathway via fimbriae protein, gingipains and lipopolysaccharides (LPS) [75–77]. However, the composition of OMVs that activate NF-κB pathway is unknown. A study demonstrated *H. p*-OMVs could inducing astrocyte reactivity through NF-κB pathway via urease and chaperonin [78]. Mun et al. found that *H. p*-OMVs stimulated gastric epithelial cells by triggering IL-8 production and NF-κB activation, presumably via OMVs-carried peptidoglycan (PG) and LPS [79]. *A. a-*OMVs induce inflammatory response in human cells by NF-κB activation, mainly due to LPS [80]. Therefore, we speculate that *P. g*-OMVs may induce the NF-κB pathway activation via virulence factors, proteins or lipid such as gingipains, PG and LPS. However, whether *P. g*-OMVs suppress ferroptosis in OSCC cells through the NF-κB signaling pathway is unclear. To answer this question, we combined NF-κB inhibitor BAY 11–7082 to investigate the functional effect of the NF-κB pathway on ferroptosis. The results revealed that the expressions of SLC7A11 and GPX4 were upregulated accompanied by the downregulation of PTSG2 and TFR by *P. g*-OMVs stimulation, the application of BAY 11–7082 reverses these findings. Our study demonstrated that *P. g*-OMVs inhibit OSCC ferroptosis via the NF-κB pathway (Figure 4). Targeting NF-κB pathway inhibition may be expected to induce the ferroptosis of OSCC. EMT has served as the key marker of tumor invasion and metastasis. An observation showed that eriocitrin inhibit EMT by triggering ferroptosis in lung adenocarcinoma cell lines A549 and H1299 [81]. Activation of EMT increases tumor motility by promoting local invasion and secondary colonization [82]. Consistent with the previous publications, our current results showed that *P. g*-OMVs promote EMT in OSCC by inhibiting ferroptosis (Figure 5), implicating that ferroptosis is involved in regulating EMT in OSCC. Hence, generating ferroptosis in tumor cells contributes to the inhibition of the malignant progression of OSCC. Tumor cells maintain their viability and proliferative capacity by modulating the metabolic reprogramming in response to changes in the tumor microenvironment (TME), resulting in the production of more reactive oxygen species and other oxidized substances within the tumor cells, and ultimately rendering tumor cells more susceptible to redox imbalance and more sensitive to ferroptosis [83]. Even though triggering ferroptosis in tumor cells has proven to be an effective anti-tumor treatment. The complex TME and tumor heterogeneity contribute to medication resistance in tumor cells, resulting in the recurrence and spread of the tumor. EMT is one of the essential mechanisms in the development of medication resistance [84]. Therefore, interfering ferroptosis-induced EMT is expected to be a promising therapeutic approach to reduce medication resistance in cancer.

Due to the invasive nature of clinical dental procedures, the oral microbiome readily induce inflammation by entering the bloodstream via inflamed gums or periodontal tissues. We previously revealed that oral microbiome *Streptococcus mutans* generates vascular inflammation to facilitate the breast cancer cell metastasize to the lungs by disrupting endothelial integrity [85]. Interestingly, Huang et al. found that *P. g*-OMVs accelerate retinopathy by destroying human retinal microvascular endothelial cells through the production of inflammatory factors and increasing vascular endothelial permeability [86]. Another study reported that *P. g*-OMVs significantly increased vascular permeability in vitro [87]. The above results suggested that *P. g*-OMVs modulate vascular endothelial cells to impair vascular integrity, thereby promoting systemic disease and tumor metastasis. This study focuses on the role of *P. g*-OMVs in mediating direct communication with tumor cells through ferroptosis and EMT to promote OSCC progression. However, further evidence is needed to determine whether *P. g*-OMVs regulate the vascular endothelial permeability of metastatic foci to accelerate secondary colonization of tumor cells. TME consists of tumor cells and various stromal cells, therefore, *P. g*-OMVs may also be involved in communication with stromal cells in addition to the direct interactions with tumor cells. Future study focusing on the communication between *P. g*-OMVs and other stromal cells, such as immune cells, to investigate the effects of *P. g*-OMVs on tumor immunomodulation is necessary. In contrast to the parent microbiome, *P. g*-OMVs potentially stimulate macrophages to produce large amounts of inflammatory factors such as tumor necrosis factor (TNF)-α, IL-6, IL-10, interferon (IFN)-β, as well as inflammasome activation and pyroptosis, resulting in persistent local inflammation [88]. Studies have shown that *P. g*-OMVs induce monocytes to selectively eliminate TNF-α and interfere with the host immune response, thereby facilitating *P. g* escape from immune surveillance [89]. One study reported that *P. g*-OMVs damaged tissue by disrupting the immune system [90]. *P. g*-OMVs could destroy periodontal tissues by acting as carriers for antigens and active proteases to trigger immune response and inflammation [91]. However, whether *P. g*-OMVs modulate the tumor immune microenvironment is unable to be assessed due to the immunodeficient mice used in the current study. Therefore, further studies carried out on immunocompetent mice should be performed.

The specific virulence factors, proteins, or lipids present in *P. g*-OMVs are unknown. Therefore, further proteomics and lipidomics studies are needed to verify the active components in *P. g*-OMVs that promote OSCC. To date, the diagnosis of tumors has been mostly based on invasive pathological tissue biopsies clinically. New diagnostic methods like blood- and saliva-based liquid biopsies are increasingly accepted by doctors and patients. Liquid-based biomarker tests may identify the presence of various diseases [92]. Compared to blood diagnostics, saliva collection is simple, non-invasive, painless, cost-effective and safe. In addition, saliva could be sampled multiple times without coagulation. Given the characteristics of the high amount of OMVs in saliva, saliva-based liquid biopsy should be advocated for tumor risk assessment in the future.

In conclusion, our study for the first time, authenticated that *P. g*-OMVs promote OSCC progression by modulating the ferroptosis-related EMT via NF-κB signaling pathway. Our research addresses the importance of oral hygiene maintenance in patients with cancer. This finding presents a new insight into the role of *P. g*-OMVs in OSCC, revealing a novel mechanism of host-pathogen interaction by which bacterial OMVs enhance cancer progression. Targeting *P. g*-OMVs inhibition may be a potential intervention for OSCC.

## Supplementary Material

Supplemental Material

## Data Availability

Data supporting the findings of the current study are available from the corresponding author upon reasonable request.
