# Supplementary material for: Porphyromonas gingivalis-OMVs promote the epithelial-mesenchymal transition of oral squamous cell carcinoma by inhibiting ferroptosis through the NF-κB pathway
Source: J Oral Microbiol. 2025 Apr 3;17(1):2482924. doi: 10.1080/20002297.2025.2482924 (PMC11980236; doi:10.1080/20002297.2025.2482924)
Supplement: Supplemental Material [file ZJOM_A_2482924_SM5491.docx]

**Supplementary materials**

| Supplement table 1 | | | |
| --- | --- | --- | --- |
| Primers used in real-time PCR. | | | |
| Gene | Full name | Primer sequence (F: forward) | Primer sequence (R: reverse) |
| Homo, GAPDH | Glyceraldehyde-3-phosphate dehydrogenase | 5’GCCGTCAACGACCCCTTCATTGA3’ | 5’GGGTGGAGTCGTACTTGAGCATGT3’ |
| Homo, Ki67 | Ki67 | 5’GAAAGAGTGGCAACCTGCCTTC3’ | 5’GTGTCACCGTTGAAGAGAGTGG3’ |
| Homo, PCNA | proliferating cell nuclear antigen | 5’CAAGTAATGTCGATAAAGAGGAGG3’ | 5’GTGTCACCGTTGAAGAGAGTGG3’ |
| Homo, E-cadherin | E-cadherin | 5’ACACCATCCTCAGCCAAGATCC3’ | 5’ACCTGACCCTTGTACGTGGTG3’ |
| Homo, N-cadherin | N-cadherin | 5’GATGTGCATGAAGGACAGCCTC3’ | 5’CGGCATACACCATGCCATCTTC3’ |
| Homo, Vimentin | Vimentin | 5’GCCAACTACATCGACAAGGTGC3’ | 5’TCTCCTCCTGCAATTTCTCCCG3’ |
| Homo, MMP9 | matrix metallopeptidase 9 | 5’CTCTATGGTCCTCGCCCTGAAC3’ | 5’ACCGGACTCAAAGGCACAGTAG3’ |
| Homo, GPX4 | glutathione peroxidase 4 | 5’AATTCGCAGCCAAGGACATCG3’ | 5’ATTCGTAAACCACACTCGGCGTA3’ |
| Homo, SCL7A11 | solute carrier family 7 member 11 | 5’CCTGCTTTGGCTCCATGAACG3’ | 5’AGAGGAGTGTGCTTGCGGACA3’ |
| Homo, PTSG2 | prostaglandin-endoperoxide synthase 2 | 5’TGCACCCACTCCCCTCTGAC3’ | 5’TGTCTCGTCAATGTCCAGCAGC3’ |
| Homo, TFR | transferrin receptor | 5’GGTTCGTACAGCAGCAGAGGTG3’ | 5’TCCACGAGCAGAATACAGCCATTG3’ |
